# Avoiding a bad apple: Insect pollination enhances fruit quality and economic value^☆^

**DOI:** 10.1016/j.agee.2013.10.032

**Published:** 2014-02-01

**Authors:** M.P.D. Garratt, T.D. Breeze, N. Jenner, C. Polce, J.C. Biesmeijer, S.G. Potts

**Affiliations:** a Centre for Agri-Environmental Research, School of Agriculture, Policy and Development, University of Reading, UK; b Norman Collett English Fruit Marketing, Norman Collett House, Kent, UK; c Faculty of Biological Sciences, University of Leeds, Leeds, UK; d Naturalis Biodiversity Center, The Netherlands

**Keywords:** Apples, Pollination, Economic valuation, Pollinators, Pollination deficit, Apple quality, United Kingdom

## Abstract

Insect pollination is important for food production globally and apples are one of the major fruit crops which are reliant on this ecosystem service. It is fundamentally important that the full range of benefits of insect pollination to crop production are understood, if the costs of interventions aiming to enhance pollination are to be compared against the costs of the interventions themselves. Most previous studies have simply assessed the benefits of pollination to crop yield and ignored quality benefits and how these translate through to economic values. In the present study we examine the influence of insect pollination services on farmgate output of two important UK apple varieties; Gala and Cox. Using field experiments, we quantify the influence of insect pollination on yield and importantly quality and whether either may be limited by sub-optimal insect pollination. Using an expanded bioeconomic model we value insect pollination to UK apple production and establish the potential for improvement through pollination service management. We show that insects are essential in the production of both varieties of apple in the UK and contribute a total of £36.7 million per annum, over £6 million more than the value calculated using more conventional dependence ratio methods. Insect pollination not only affects the quantity of production but can also have marked impacts on the quality of apples, influencing size, shape and effecting their classification for market. These effects are variety specific however. Due to the influence of pollination on both yield and quality in Gala, there is potential for insect pollination services to improve UK output by up to £5.7 million per annum. Our research shows that continued pollinator decline could have serious financial implications for the apple industry but there is considerable scope through management of wild pollinators or using managed pollinator augmentation, to improve the quality of production. Furthermore, we show that it is critically important to consider all production parameters including quality, varietal differences and management costs when valuing the pollination service of any crop so investment in pollinator management can be proportional to its contribution.

## Highlights

• Insect pollination affects not only the quantity of apple production but also the quality.
• The value of insect pollination to UK apple production may have previously been underestimated.
• UK apple production could be significantly improved through management of insect pollination.
• It is important to consider variety when valuing ecosystem services such as pollination to agricultural production.

## 1 Introduction

Insect pollinators play a fundamental role in the production of many fruits, vegetables and field crops (Klein et al., 2007) and numerous studies have valued insect pollination as an ecosystem service for agricultural food production at both global (Gallai et al., 2009; Winfree et al., 2011) and national scales (Smith et al., 2011). There is, however, increasing evidence of global and localised declines in the abundance and diversity of both managed and wild insect pollinators (Biesmeijer et al., 2006; vanEngelsdorp et al., 2008; Potts et al., 2010a,b; Cameron et al., 2011) threatening the stability of this ecosystem service. Understanding the economic benefits of an important agricultural ecosystem service such as crop pollination by insects is fundamental to sustainable food production and farm management. On the one hand, valuation allows potential consequences of continued insect pollinator decline for food production and food security to be understood, on the other hand it can illustrate how appropriate management of insect pollination services can reduce production risks and increase rewards by addressing pollination deficits within cultivated areas (Abson and Termansen, 2011).

Apple (*Malus domestica*) is one of the most important fruit crops globally, with 2010 production across 93 countries worth US$64bn (FAOStat, 2013). Apple cultivars are self-incompatible to varying extents, requiring pollen transfer from another “polliniser” cultivar to set fruit in marketable quantities (Delaplane and Mayer, 2000). Insects, such as bees and hoverflies, are the predominant pollination vector for apples and thus their activity in orchards is essential for apple production globally (Free, 1964; Delaplane and Mayer, 2000; Klein et al., 2007). Experimentally increasing insect pollinator numbers in apple orchards has shown improved fruit set and yield (Stern et al., 2001; Ladurner et al., 2004) but the influence of insect pollination on the quality of apple production in terms of size, shape, marketability and storability is less well understood. There is some evidence that levels of pollination affect seed number with associated impacts on size and calcium concentration (Brookfield et al., 1996; Volz et al., 1996; Buccheri and Di Vaio, 2004; Matsumoto et al., 2012) but direct links between insect pollination and apple quality are equivocal.

Within the UK, apple production is a major component of the horticultural sector, occupying 82% of orchard fruit area as of 2010 (DEFRA, 2012b). Of this, dessert apple production is the most valuable component with a net value of £63 m nationally. Following Smith et al. (2011), the value of pollination services to this production stands at £58 m. However, this estimate is based upon the dependence ratio method, a metric of the theoretical loss of yield resulting from an absence of pollinators (Gallai et al., 2009). Dependence ratios are drawn from primary literature that utilise a range of cultivars and experimental designs and usually focus upon fruit set only, neglecting factors such as market quality and fruit abortion that will affect the final market output, potentially overestimating service values (Bos et al., 2007; Winfree et al., 2011). Furthermore, knowledge of the influence of pollination services on quality as well as yield is important in terms of the economic value of insect pollinators to apple production and has implications for storage and waste reduction. Identifying the production value that is currently limited by sub-optimal pollination is also necessary and potentially provides an economic benchmark indicating how much could be sensibly invested in management of insect pollination services to address these deficits. Such pollination deficits have been found in several tree crops including apples (Brookfield et al., 1996; Volz et al., 1996; Pritchard and Edwards, 2006; Holzschuh et al., 2012).

In order to more accurately estimate the full economic benefits of pollination to production of an economically important crop, we have utilised field studies and a novel bioeconomic model to examine the influence of pollination services on farmgate output of two important varieties of apples grown in the UK; Gala and Cox. In so doing we have: (1) quantified the influence of insect pollination on the yield and quality of the two most widespread varieties of UK apples; (2) through the use of supplementary pollination, determined if UK apple yield and quality may be limited by sub-optimal insect pollination (i.e. highlighted deficits) and; (3) estimated the economic value of insects to apple production and potential profitability and highlighted how important it is to consider variety and quality when valuing crop pollination services.

## 2 Materials and methods

### 2.1 Sites

In three distinct geographical locations, separated by at least 10 km, in the apple growing area of Kent, UK, an orchard of Gala and Cox were selected for experimental trials in 2012. Each of the six orchards was conventionally managed, incorporating the use of chemical pesticides and fertilisers, in common with the vast majority of apple production in this region. The orchards ranged from 4 to 16 years of age were surrounded by plantations of other varieties of apple and at least 1.2 ha in size. Specific polliniser varieties had not been planted at any of the orchards; they were located next to blocks of compatible varieties which were Cox and Gala at five of the orchards and Cameo at one of the Cox orchards.

### 2.2 Pollination treatments

Within each of the six orchards, 3 centrally located rows were identified each with at least one row of trees between them. In each row 10 trees, with two trees between them and at least 25 m from the orchard edge, were selected. Despite some variation in the size and shape of experimental orchards, this sampling distribution allowed for a good representation of treatment effects across the orchards and reduced edge effects. Approximately one to two weeks before flowering, four inflorescences on the same side of each tree, to avoid confounding effects of shade and microclimate, were selected and randomly assigned to one of three treatments. Thus 720 inflorescences were manipulated for this study. One was to receive the hand pollination treatment, one was to remain pollinated by the natural insect community and remaining two were to have insect pollinators excluded entirely. More inflorescences received the pollinator exclusion treatment so that, despite lower predicted fruit set, enough apples would still set to establish apple quality in the absence of insect pollination. The hand pollinated and pollinator excluded inflorescences were then covered with a PVC mesh bag with a mesh size of 1.2 mm^2^, these are wind and rain permeable but exclude visitation by insects, bags were included on hand pollination treatments so the source of pollen to experimental flowers could be controlled. At peak flowering (early May) each orchard was visited and on hand pollinated inflorescences, the bag was removed and all open flowers were pollinated using a paint brush. For each orchard, pollen was taken from dehisced anthers on flowers in the neighbouring apple plot, which in all cases was an appropriate polliniser for Gala and Cox. When flowering had finished at all sites, bags were removed and inflorescences were marked with coloured cable ties and string so they could be located for harvest. Bagging and supplemental pollination experiments such as this have been utilised in other pollination studies of apples (Volz et al., 1996) and other tree fruit (Soltesz, 1997; Isaacs and Kirk, 2010).

### 2.3 Fruit set measurements

Prior to commercial thinning carried out on some of the orchards (early July), a visit was made to each site. For each experimental inflorescence, the number of set apples was recorded. The apples on each branch which included any experimental inflorescences were then thinned according to standard industry practice; this sometimes included the removal of apples from experimental inflorescences so no more than two remained on any one inflorescence. Any apples removed from experimental inflorescences during thinning were taken back to the laboratory for seed number analysis.

### 2.4 Quality measurements

All apples from experimental inflorescences were collected one day to a week before commercial harvest at each of the orchards (early September for Cox, mid September for Gala). Apples were bagged individually by treatment, tree, row and orchard and taken back to the laboratory for quality assessment. Within 5 days of harvest, quality measures had been taken from 531 apples and seeds had been counted in 735 apples.

Quality measures included; fresh weight, taken on a Mettler Toledo balance sensitive to the nearest 0.1 g; maximum width, measured using callipers sensitive to 0.1 mm; firmness in kg/cm, taken using a Silverline penetrometer and percentage sugar concentration or Brix, using a Hanna refractometer. Apples were also scored for shape, either classified as ‘normal’, or ‘deformed’ if there was any shape irregularity. Size, shape, sugar content and firmness are all measures used by the apple processing industry to understand the quality of apples produced. Larger apples with an even shape are preferred and high sugar content and firmness is considered desirable and gives improved taste. Additional data on the seed number per apple was taken from apples removed at harvest and on the visit made to orchards for apple thinning. In order for the economic value of pollination to apple production to be calculated, apples were classed using parameters utilised in the industry (Jenner, 2013, pers. comm.). Apples were classified as class 1 or 2 based on size and shape. Class 1 apples are those with no shape deformities and a maximum width greater than 60 mm, all other apples were class 2.

The mineral content of apples can have marked effects on quality parameters such as storability and resistance to disease (Conway et al., 2002). Given the clear effects of insect pollination on Gala quality, for a sub set of the Gala apples, mineral analysis was carried out. Apples from those trees with at least one open pollinated, hand pollinated and pollinator-excluded apple set were sent for mg/100 g measurements of calcium, phosphorus, nitrogen, potassium, magnesium, boron and zinc. This means 87 apples from 29 trees were involved in mineral analysis.

### 2.5 Economic valuation

Market value of apple production per hectare depends on several factors, some of which are directly influenced by pollination services; higher fruit set and greater weight result in greater overall output and quality parameters, such as size and shape, affect the price paid per kilogram. As such, the market value of pollination services, in terms of added market output in a given year is a product of the added quality and quantity of apples, estimated in this study as:

(1) $$PV_{c}=(V_{cOPEN}-V_{ci})A_{c}$$

where *PV*_*c*_ is the output added by insect pollination services in cultivar *c* across the UK, *V*_*cOPEN*_ and *V*_*ci*_ are the total economic output per hectare of cultivar *c* under open pollination and either closed (when considering current value) or hand pollination (when considering deficits) treatments and *A*_*c*_ is the total area of cultivar *c* in 2010, taken from DEFRA (2012b,c). For each treatment group total economic output (*V*_*ct*_) was calculated as:

(2) $$V_{ct}=V_{ct1}+V_{ct2}-\Delta C_{t}$$

where *V*_*ct*1_ and *V*_*ct*2_ are the total value of class 1 and class 2 apples produced under treatment *t*. Δ*C*_*t*_ represents percentage changes in the thinning costs per hectare under treatment t based on industry data (Jenner, 2013, pers. comm.), see Appendix 1 for details. Other producer costs are quantity insensitive and are not considered. The economic output of quality class *i* under treatment *t* (*V*_*cti*_) is:

(3) $$V_{cti}=P_{cti}\times O_{cti}$$

where *P*_*cti*_ is the price/kg of apples of cultivar *c* of quality class *i* taken from DEFRA (2013) and *O*_*cti*_ is the total quantity of apples of quality class *i* produced. *O*_*cti*_ could not be derived directly from the limited sample data used in this study and instead was estimated as:

(4) $$O_{cti}=Y_{c}\times S_{ct}\times Q_{cti}\times W_{cti}$$

where *Y*_*c*_ is the average national yield/ha of cultivar *c* in 2010 derived from (DEFRA, 2012b), for Gala, which is not independently reported, the yield/ha of late season apples was used as a proxy. *S*_*ct*_ is the percentage total fruit set compared to open pollination under treatment *t*, *Q*_*cti*_ is the percentage of sampled apples in quality class *i* from treatment *t* and *W*_*cti*_ is the percentage difference in average weight of apples of quality classification *i* in treatment *t* compared to open pollination. To capture rates of abortion and thinning, *S*_*ct*_ was calculated based on the final harvested yield of marketable fruit compared with the maximum post-thinning yield of 2 apples per inflorescence:

(5) $$S_{ct}=\frac{F_{ct}}{2(B_{ct})}$$

where *F*_*ct*_ is the final total number of fruits harvested divided by twice the number of inflorescences studied in treatment group *t* (*B*_*ct*_) to represent maximum potential yield. These findings were compared with two more typical, quality independent, dependence ratio analyses based on percentage changes in pre-thinning fruit set and harvest yield (Appendix 1). The average value of the two-cultivar specific dependence ratios was used to estimate a cultivar independent value. These dependence ratios were then multiplied by the average price/kg of the two classes to produce estimates of value per hectare. For the cultivar independent analysis the average price of both classes was used as a price/kg and the total area of both cultivars used to estimate total output value.

### 2.6 Statistical analysis

Generalised linear mixed effects models were used to investigate pollination treatment effects on fruit set and seed number. Pollination treatment was a fixed effect and inflorescences nested within trees, nested within rows, nested within orchards were random effects, thus accounting for any potential variation between orchards in terms of management and local environmental factors. Seed number is a count so Poisson error structure was defined and fruit set is a proportion so a binomial error structure was used. Width, weight, firmness and Brix were all normally distributed so a linear mixed effects model was used with the same fixed and random effects as for the generalised linear mixed effects model.

Gala apple mineral concentration was analysed using a linear mixed effects model. For each mineral a Box–Cox test was used to determine the most appropriate transformation and subsequently calcium, phosphorus, nitrogen and zinc were log transformed prior to analysis. Boron and magnesium were square root transformed and potassium remained untransformed. All analyses were carried out in R version 2.14.1.

## 3 Results

### 3.1 Apple yield

**Fig. 1:**
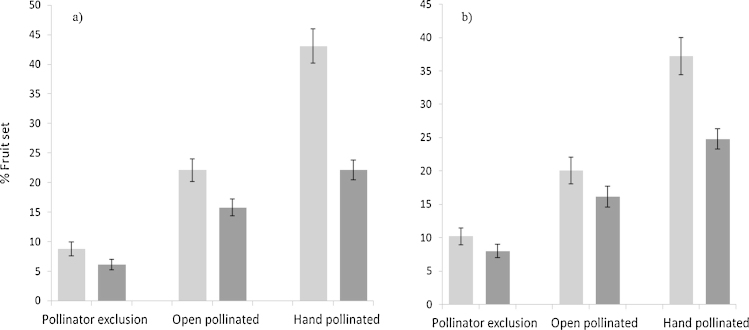
The effect of pollination treatment on the fruit set of Cox (a) and Gala (b) apples at the time of thinning () and at harvest (◆), mean ± S.E.M.

The fruit set of Cox (Fig. 1a) was affected by pollination treatment with significantly greater fruit set following hand pollination compared to open pollinated blossoms (*Z*_2,356_ = 7.59, *P* < 0.0001) which in turn set more fruit than pollinator excluded blossoms (*Z*_2,356_ = 7.30, *P* < 0.0001). This was the same for fruit remaining at harvest (Fig. 1a) following thinning and stochastic losses, with hand pollinated fruit numbers higher than open pollinated (*Z*_2,353_ = 2.19, *P* = 0.029) which was higher than pollinator excluded blossoms (*Z*_2,353_ = 6.21, *P* < 0.0001). The same treatment effects on fruit set were found for Gala (Fig. 1b), hand pollinated blossoms set more fruit than open pollinated blossoms (*Z*_2,354_ = 5.89, *P* < 0.0001) which again set more than pollinator excluded blossoms (*Z*_2,354_ = 5.72, *P* < 0.0001). The same relationship was found for apple number at harvest (Fig. 1b) with hand pollinated greater than open pollinated (*Z*_2,353_ = 3.18, *P* = 0.0015) which was greater than pollinator excluded (*Z*_2,353_ = 5.18, *P* < 0.0001).

### 3.2 Apple quality

**Fig. 2:**
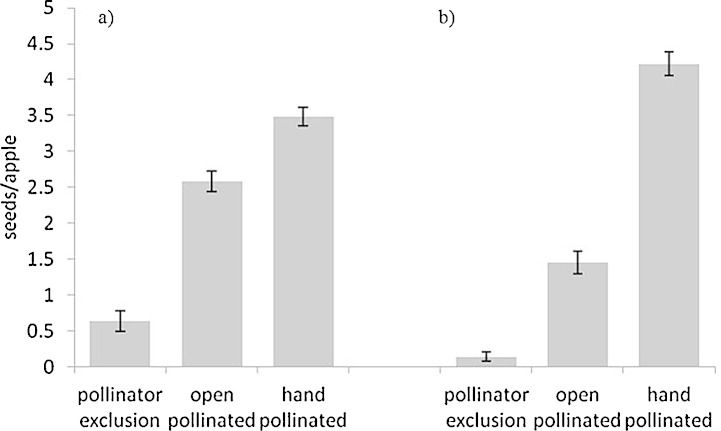
The effect of pollination treatment on the seed set of Cox (a) and Gala (b) apples, mean ± S.E.M.

The number of seeds per apple for Cox (Fig. 2a) was significantly affected by treatment with a greater seed number per apple in hand pollinated than open pollinated fruit (*Z*_2,364_ = 4.15, *P* < 0.0001) which in turn had greater seed numbers than pollinator excluded fruit (*Z*_2,364_ = 8.78, *P* < 0.0001). The same significant relationship was found for Gala (Fig. 2b) with hand pollinated greater than open pollinated (*Z*_2,371_ = 10.88, *P* < 0.0001) which was greater than pollinator excluded fruit (*Z*_2,371_ = 8.11, *P* < 0.0001).

Pollination treatment had a significant effect on the size, weight and firmness of Cox apples (Table 1). Open pollinated apples were significantly larger and heavier than hand pollinated apples. Furthermore open pollinated apples were significantly softer than both pollinator excluded and hand pollinated apples. A contrasting effect of pollination treatment on Gala was seen (Table 1). Hand pollinated apples were significantly larger than open pollinated apples which were in turn significantly larger and heavier than those with pollinators excluded. Pollinator excluded apples were significantly firmer than open pollinated and hand pollinated apples.

Pollination treatment significantly affected the concentration of calcium, magnesium and zinc in experimental apples (Table 2). In all cases, hand pollinated apples had a significantly lower concentration than pollinator excluded apples. For calcium and magnesium, concentrations in hand pollinated apples were also significantly lower than in open pollinated fruit which were also significantly lower than concentrations found of magnesium and zinc in pollinator excluded fruit.

### 3.3 Economic output

In both cultivars, the market output extrapolated from the analysis was substantially higher in open compared to closed treatments, adding £11,900 (Cox) and £14,800 (Gala) per hectare (IPV/ha). In total this is equivalent to £36.7 m in added output across the UK area of these cultivars (national total – Table 3). Hand pollination demonstrated substantially lower effects on total market production in Cox than Gala. In the former, hand pollination only slightly increased fruit number while weight and proportion of class 1 apples were similar to the closed treatment. The resultant higher thinning costs potentially reduce national market output by £0.3 m (total deficit) or ∼£146 per hectare (deficit/ha). By contrast, higher fruit set and greater proportion of class1 fruit in hand pollinated gala suggests an output deficit of ∼£5.7 m (£6469/ha).

Estimates of market output value derived from more standard dependence ratio metrics, based on differences in either initial fruit set (DRy) or final production quantity, independent of quality considerations (DRp), were substantially lower than the inclusive estimates above (Table 4), underestimating the market value of pollination services to these cultivars by £3.2–5.9 m nationally. The very high numbers of initial flowers resulted in an unrealistically high maximum yield upon which fruit set based dependence ratios were made. In both cases the insensitivity to the price difference between apple classes drives under estimation, even though DRp is close to the true proportion of market yield lost. Notably the prices per kg of class 1 apples are much higher than the average prices estimated from DEFRA data (£0.62/kg, estimated from (DEFRA, 2012b), which are the basis for past valuation studies (Carreck and Williams, 1998; Smith et al., 2011). Making these estimates independent of cultivar further underestimates the market value of pollination service due to the lower prices paid for Gala.

## 4 Discussion

The fundamental importance of insect pollination to UK apple production is evidenced by the reduction in fruit set following pollinator exclusion in both apple varieties involved in this study. Effects on apple quality by contrast are variety specific with only Gala showing any significant improvement in size and weight following insect pollination. These varietal differences demonstrate how important it is to consider multiple varieties when trying to understand insect pollination and top fruit production. Positive relationships between seed number and quality parameters including size, evenness of shape and mineral content have been found in other varieties (Brookfield et al., 1996; Volz et al., 1996; Buccheri and Di Vaio, 2004; Matsumoto et al., 2012). The positive effect of insect pollination on seed number in Cox found in this study suggests that Cox might be unusual in demonstrating no relationship between seed number and apple size and positive effects of insect pollination on apple quality may be the norm.

In the present study, the mineral concentration of Gala apples was significantly affected by pollination treatment with Ca, Mg and Zn concentrations higher in pollinator excluded, then open pollinated followed by hand pollinated apples. The contrasting effect of treatment on apple weight suggests a possible dilution effect and concentrations of these minerals are reduced in larger apples despite the larger apples typically having higher seed numbers. This contradicts findings in some other apple studies (Brookfield et al., 1996; Volz et al., 1996; Buccheri and Di Vaio, 2004) but may be an artefact of varietal differences. It is important to note that under all treatments, the concentrations of Ca, commonly measured by apple growers because of its potential impact on long term storage, was well above the 4.5 mg/100 g typically used as a deficiency threshold (Jenner, 2013, pers. comm.).

The market value of insect pollination to apple production is clear for both Cox and Gala, adding >£11,000 and >£14,000 in additional output per hectare, respectively. Our data also shows, however, that there is evidence of potential economic pollination deficits for Gala where pollination affects both yield and quality. Under optimal pollination conditions, market output of Gala would be increased by £6500/ha. Current levels of investment in pollination services, either through management of wild populations or introduction of managed species, remains largely unknown and as such the importance of insect pollinators may be undervalued. The continued decline of insect pollinators could have serious ramifications for the apple industry and the £37 million service they provide to Cox and Gala production. Furthermore, the large scope for potential improvement in market output for Gala, highlights the potential for investment in management to boost pollination services.

In many countries (Delaplane and Mayer, 2000) and historically in the UK (Carreck et al., 1997), honey bee colonies have been utilised to facilitate pollination in apple orchards and indeed sequential introduction has shown significant increases in apple yield can be achieved (Stern et al., 2001). There is also increasing evidence for the importance of wild bees for the pollination of many fruit crops (Garibaldi et al., 2013) and management to exploit this resource is also possible. Planting additional floral resources which flower at different times to apple can improve the reproductive success of apple visiting solitary bees (Sheffield et al., 2008). Furthermore, establishment or the preservation of existing natural or semi-natural areas in or around orchards can increase the abundance and diversity of wild pollinators with associated benefits to fruit production (Chacoff and Aizen, 2006; Garibaldi et al., 2011; Watson et al., 2011; Holzschuh et al., 2012). This study presents a maximum potential gain following optimal pollination but this maximum may not be achievable through management of pollinators alone. In practice it is likely a case of diminishing returns on production with improvements in insect pollination. It would take a cost benefit analysis, incorporating the cost of various management practices and their potential benefits to pollination service, to justify the implementation of a management strategy. The impact of any pollinator management would be context dependent and moderated by many external factors associated with individual orchards, including local landscapes and agricultural management practices. An important avenue of future research is to understand, in the first instance how these external factors influence the level of pollination service and the extent of pollination deficits, but also how they might influence the value of deficit mitigation strategies. The extent of the pollination deficits to UK Gala apple production in 2012 stands at £5.7 million, this certainly justifies further research into optimising orchard pollinator management.

Current crop research has increasingly highlighted both pollinator dependent yield limitation (Pando et al., 2011; Magalhaes and Freitas, 2013) and the benefits of adequate pollination services on crop quality, (Chagnon et al., 1993; Roldan Serrano and Guerra-Sanz, 2006; Roselino et al., 2009; Isaacs and Kirk, 2010; Bommarco et al., 2012). The findings of our research represent several improvements in the economic valuation of pollination services and highlight many of the shortfalls from assumptions made in past studies. Foremost, the study emphasises the importance of accounting for quality, in addition to yield variation, as a parameter of economic output. Although the yield based dependence ratios of past studies may have been similar (Carreck and Williams, 1998) or greater (Smith et al., 2011) than the estimated proportion of market output lost without pollination in this study, by failing to account for quality driven price variation they have likely underestimated the value of pollination services. Secondly, this study demonstrates the importance of accounting for cultivar variations in pollination service benefit when estimating economic value. Not only are the yield responses lower than most other studies (Allsopp et al., 2008) but the total value of services is much greater in Gala than Cox. As such the cultivar independent dependence ratio analysis also underestimates the total benefits. Finally, the methods used in this study allow for estimations of service deficits by highlighting the differences between observed and potential market benefits, in this instance totalling some £6 m.

Although comprehensive, the market benefits estimated in this study may still be distorted by several unobserved factors. This study has inherently assumed that only pollination limits yields, however other factors such as pest damage and soil nutrients may limit yields to an equal or greater extent (Bommarco et al., 2013). Limitations in these services may explain the lower ratio of class 1 apples in hand pollinated Cox compared with open pollinated treatments if, for example, nutrients were too limited to allow an increased number of apples to fully develop. If the ratios of quality classes were identical between open and hand pollinated treatments, the added value would be more than sufficient to compensate for the added thinning costs. By a similar measure the benefits of pollination services may be influenced by limitations in other aspects of crop production, such as soil fertility (Bommarco et al., 2013). Different polliniser cultivars may also influence fruit set depending on the genetic compatibility of the two varieties (Matsumoto et al., 2007) which may in turn influence yield and quality.

The impacts of pollination services may fluctuate between years depending on inter-annual variation in plant resource use (Garibaldi et al., 2011) but it is not only ecological fluctuations that may affect pollination service quantification but economic and demand variability in the supply chain will also have an effect. In this study, farmgate price was used to estimate benefit, however farmgate prices for apples represent only 42% of the final price paid by consumers and secondary consumers (DEFRA, 2012a) and are in turn influenced by total market supply and demand. Consequently, if pollination services were completely lost, prices at the farm gate may spike, reducing farmer losses but causing a decrease in consumer welfare as the added costs are passed on (Southwick and Southwick, 1992; Gallai et al., 2009). However, this relationship will be further complicated by the additional supply of imports and the availability of apples of different quality classes, data for which are not presently available. The estimated economic value of benefits to apple production in this study should therefore be taken as a quantification of output lost rather than a full valuation of pollination services.

## 5 Conclusion

Insect pollination is essential to apple production in the UK, both in terms of yield and quality, but only by understanding the true value of this service to growers can sensible and economically justifiable management decisions be made. In the first instance, to arrest potential pollinator declines, but also to manage pollination services to increase the value of production above current levels. It is becoming increasingly clear that insect pollination service needs to be considered as one of the many ecosystem and agronomic inputs a grower must manage and it needs to be integrated into the whole production and marketing system. The methodologies used in the study to incorporate quality parameters into the valuation of pollination services and thus understand its importance as an agricultural input could be adapted and utilised for other insect pollinated crops. From this the value of insect pollination to agriculture as a whole will become clearer. Such information can underpin farm management and policy decisions focussed on promoting insect pollination services so effective crop production can be maintained and improved in the face of ongoing environmental change. Understanding the multiple benefits of an ecosystem service, such as pollination, is critical for ensuring food security, as it is not only the yield but the quality and value of produce which are important.

## Figures and Tables

**Table 1:** Effect of pollination treatment on Gala and Cox quality measures (mean ± S.E.M.). *F* and *P* value from linear mixed effects models shown.

Variety	Measure	Pollinator exclusion	Open pollinated	Hand pollinated	*df*	*F* value	*P* value	Significant differences
Cox	Width (cm)	68.1 ± 0.8	69.2 ± 0.8	66.2 ± 0.8	100	4.29	0.017	Open > hand
	Weight (g)	140.7 ± 4.7	148.8 ± 4.4	133.1 ± 4.5	100	4.10	0.019	Open > hand
	Sugar (%)	11.7 ± 0.2	11.4 ± 0.1	11.7 ± 0.1	100	1.65	0.195	
	Firmness (kg/cm)	9.7 ± 0.2	9.0 ± 0.1	9.6 ± 0.1	100	7.37	0.001	Open < hand, closed

Gala	Width (cm)	57.4 ± 1.0	62.1 ± 0.5	65.5 ± 0.5	108	50.55	<0.0001	Hand > open > closed
	Weight (g)	100.9 ± 4.0	119.9 ± 2.8	131.8 ± 2.9	108	33.08	<0.0001	Hand, open > closed
	Sugar (%)	11.7 ± 0.2	12.0 ± 0.2	11.8 ± 0.1	108	1.06	0.350	
	Firmness (kg/cm)	11.4 ± 0.4	9.9 ± 0.2	9.6 ± 0.1	108	25.66	<0.0001	Hand, open < closed

**Table 2:** Effect of pollination treatment on Gala mineral content (mean ± S.E.M.). *F* and *P* value from linear mixed effects models shown.

Mineral (mg/100 g)	Pollinator exclusion	Open pollinated	Hand pollinated	*df*	*F* value	*P* value	Significant differences
Ca	9.1 ± 0.44	9.1 ± 0.40	7.6 ± 0.29	55	5.98	0.004	Hand < open, closed
P	8.7 ± 0.29	8.3 ± 0.26	8.0 ± 0.25	55	3.12	0.052	
N	41.5 ± 2.10	38.6 ± 1.63	37.1 ± 2.13	55	2.28	0.112	
K	101.1 ± 3.16	98.4 ± 2.80	98.2 ± 2.26	55	0.65	0.528	
Mg	5.8 ± 0.16	5.4 ± 0.13	4.9 ± 0.12	55	25.29	<0.0001	Hand < open < closed
B	0.25 ± 0.011	0.26 ± 0.013	0.24 ± 0.009	55	0.25	0.783	
Zn	0.043 ± 0.003	0.036 ± 0.002	0.033 ± 0.002	55	10.27	<0.0001	Hand, open < closed

**Table 3:** Value of present and potential pollination services to Cox and Gala apples at a hectare and national scale.

	Cox	Gala
Price/kg class 1(£)	0.86	0.77
Price/kg class 2 (£)	0.50	0.52
Total value/ha (£000)	£19.6	£22.9
Total IPV/ha (£000)	£11.9	£14.8
National total (£000)	£23,740.5	£12,965.5
Deficit/ha (£000)	−£0.1	£6.5
Total deficit (£000)	−£291.5	£5679.7

*Key*: Total value/ha – the present total value of market output per hectare estimated from the open pollination treatment; total IPV/Ha – the total insect pollination service value per hectare; the difference in output from the open and closed treatments; national total – the total value of insect pollination services to the crop across the UK; deficit/ha – the difference in total IPV between hand and open pollination treatments representing the potential value of production lost due to service limitation; total deficit – the total value of production deficits across the UK.

**Table 4:** Estimates of the economic value of pollination services at hectare and national scales using quantitative and cultivar independent dependence ratio analyses.

	Cox		Gala		cv. independent	
	DRy	DRp	DRy	DRp	DRy	DRp
	59.8%	63.1%	51.6%	59.1%	55.7%	61.1%
IPV/ha (£000)	£9.4	£10.0	£9.8	£11.1	£9.7	£10.6
National total (£000)	£18,802.1	£19,811.3	£8561.9	£9805.5	£27,809.9	£30,482.3
Difference (£000/ha)	−£2.9	−£2.0	−£5.0	−£3.2	−£2.2/−£5.1	−£1.3/−£4.1
Total difference (£000)	−£5874.1	−£3929.2	−£4403.7	−£3159.9	−£8896.1	−£6223.4

*Key*: cv. independent – cultivar independent estimates, developed using average DR values, prices and outputs/ha; DRy – the proportion of pre-thinning yield lost in the absence of pollination services; DRp – the proportion of total crop production (fruit set × weight) lost without pollination services; IPV/ha – total insect pollination service value per hectare; national total – the total value of insect pollination services to the crop across the UK. For the cv. independent column this is based upon the sum area of both cultivars; difference/ha – the difference between per hectare estimates using these dependence ratios and the values per hectare estimated in Table 3. Values are given for Cox and Gala, respectively in the cv. independent column; total difference – the difference between total value estimates across the whole area of crop. For the cv. independent column this is based on the difference between the estimated total and the sum of the national total estimates in Table 3.
