# Supplementary material for: Avoiding a bad apple: Insect pollination enhances fruit quality and economic value
Source: Agric Ecosyst Environ. 2014 Feb 1;184(100):34–40. doi: 10.1016/j.agee.2013.10.032 (PMC3990452; doi:10.1016/j.agee.2013.10.032)
Supplement: Supplementary file 1 [file mmc1.docx]

**Appendix 1** Alternative Dependence Ratio Metrics

To illustrate the importance of considering quality parameters within the value of pollination services to crops, two more typical dependence ratio analyses were conducted. These analyses multiply the total output by a metric of output produced in the absence of pollination (the dependence ratio).

$$V_{c}=MP_{c}\times O_{ct}$$

$$PV_{c}=V_{c}\times DR_{c}$$

Where *V_c_* is the total value of output of cultivar *c*, calculated as the median price/kg of both apple classes (*MP_c_*) multiplied by total output/ha and *O_ct_* is the total quantity of apples produced under treatment *t* (open, closed, hand). *DR_c_* represents the dependence ratio of cultivar c. The first dependence ratio type, *DR_y_* is based on the differences in fruit set between treatments, capturing only the effect on initial, pre-thinning fruit set. This is calculated as:

$$DR_{y}=\frac{T_{ct}}{T_{cOPEN}}$$

*DR_y_* is the insect pollination dependence ratio derived from differences in pre-thinning fruit set, equal to the percentage of flowers that set fruit under either closed (*T_cCLOSED_*) or hand pollination (*T_cHAND_*) treatments (*t*) divided by the percentage set under open pollination (*T_cOPEN_*) before thinning.

$$DR_{o}=\frac{U_{ct}}{U_{cOPEN}}$$

$$U_{ct}=Y_{c}\times S_{ct}\times W_{ct}$$

The second dependence ratio metric, *DR_p_*, is the insect pollination dependence ratio derived from differences in final production between treatments, considering the total weight of crop produced (*U_ct_*). *U_ct_* is the product of the average national yield/ha of cultivar c in 2010 (*Y_c_*), derived from DEFRA (2012b), the percentage total fruit set compared to open pollination (*S_ct_*) and the percentage average weight of apples in treatment t compared with open pollinated apples (*W_cti_*).
